# Complete Genome Sequence of Citrobacter freundii Myophage Miller

**DOI:** 10.1128/genomeA.01425-14

**Published:** 2015-01-15

**Authors:** Kyuwon Hwang, Adrian J. Luna, Adriana C. Hernandez, Gabriel F. Kuty Everett

**Affiliations:** Center for Phage Technology, Texas A&M University, College Station, Texas, USA

## Abstract

Citrobacter freundii is a Gram-negative, opportunistic pathogen that can be fatal to newborns or immunocompromised patients. Bacteriophages against this bacterium can be useful for therapeutic purposes. Here, we describe the complete genome and the key features of the pseudo T-even C. freundii bacteriophage Miller.

## GENOME ANNOUNCEMENT

Citrobacter freundii is a Gram-negative ubiquitous bacterium. It is a member of the family Enterobacteriaceae and is associated with fatal neonatal meningitis and various nosocomial infections (1). The treatment of such conditions is difficult due to the broad range of antibiotic resistance expressed by many strains of the bacterium (2). Moreover, it has been proposed that the C  freundii may play a role in disseminating antibiotic resistance genes to other microbial communities in soil (3). Bacteriophages against C. freundii could be used to quell the growing problem of this pathogenic bacterium.

Bacteriophage Miller was isolated from a sewage sample collected in Bryan, TX, USA. Phage DNA was sequenced in an Illumina MiSeq 250-bp paired-end run with a 550-bp insert library at the Genomic Sequencing and Analysis Facility at the University of Texas (Austin, TX, USA). Quality controlled, trimmed reads were assembled to a single contig at 30.1 fold coverage using Velvet version 1.2.10. Contigs were confirmed to be complete by PCR. Genes were predicted using GeneMarkS (4) and corrected using software tools available on the Center for Phage Technology (CPT) Galaxy instance (https://cpt.tamu.edu/galaxy-public/). Morphology was determined using transmission electron microscopy performed at the Texas A&M University Microscopy and Imaging Center.

Miller is an RB43-like, morphotype A2 myophage that shares 88.2% homology with RB43 (NC_007023) as determined by Emboss Stretcher (5, 6). The genomic differences are largely attributed to hypothetical conserved/novel genes of unknown function. Unlike RB43, which has a fused *rIIa* and *rIIb* gene, Miller has separate *rIIa* and *rIIb* genes that share an overlapping start/stop codon. As with RB43 and other pseudo T-even phages, the genome of Miller is opened to *rIIb* (7). Miller has a 178,171-base pair genome, a coding density of 95.6%, 43.13% G+C content, and 277 predicted genes. The G+C content is comparable to RB43 (43.2%). Of the 277 predicted coding sequences, 170 are hypothetical conserved/novel, and 99 were given a putative function based on BLASTp and InterPro analysis (8, 9). Miller encodes one homing endonuclease with an AP2 DNA-binding domain compared to the three AP2 domain containing HNH homing endonucleases of RB43. Like RB43, Miller encodes a single tRNA gene. In RB43, the tRNA is an Ile where as in Miller, it is a Met as has been reported in RB43-like Escherichia coli phage Lw1 (7).

Overall, the genome of Miller is syntenic with that of RB43. Pseudo T-even-like genes encoding the proteins for biosynthesis, DNA replication, recombination, morphogenesis, DNA packaging, and lysis were annotated. A T-even-like lysis cassette was identified including a class III holin, inner and outer membrane spanins, an rI-like antiholin, and an endolysin. Unlike T4, which encodes a muraminidase (EC 3.2.1.17); the endolysin of Miller is a peptidase (EC 3.4.16.4) as with other RB43-like phages. An additional peptidoglycan-binding protein was identified by its LysM domain (IPR018392), but the role of this protein in the page infection is unknown.

### Nucleotide sequence accession number.

The genome sequence of phage Miller was contributed as accession no. KM236237 to GenBank.

## References

[1] Badger JL, Stins MF, Kim KS 1999 *Citrobacter freundii* invades and replicates in human brain microvascular endothelial cells. Infect. Immun. 67:4208–4215. 10417193 10.1128/iai.67.8.4208-4215.1999 PMC96726

[2] Yim G, Kwong W, Davies J, Miao V 2013 Complex integrons containing qnrB4-ampC (bla(DHA-1)) in plasmids of multidrug-resistant *Citrobacter freundii* from wastewater. Can. J. Microbiol. 59:110–116. doi:10.1139/cjm-2012-0576. 23461518

[3] Srinivasan V, Nam HM, Sawant AA, Headrick SI, Nguyen LT, Oliver SP 2008 Distribution of tetracycline and streptomycin resistance genes and class 1 integrons in Enterobacteriaceae isolated from dairy and nondairy farm soils. Microb. Ecol. 55:184–193. doi:10.1007/s00248-007-9266-6. 17701242

[4] Besemer J, Lomsadze A, Borodovsky M 2001 GeneMarkS: a self-training method for prediction of gene starts in microbial genomes. Implications for finding sequence motifs in regulatory regions. Nucleic Acids Res. 29:2607–2618. doi:10.1093/nar/29.12.2607. 11410670 PMC55746

[5] Ackermann HW, Krisch HM 1997 A catalogue of T4-type bacteriophages. Arch. Virol. 142:2329–2345. doi:10.1007/s007050050246. 9672598

[6] Myers EW, Miller W 1988 Optimal alignments in linear space. Comput. Appl. Biosci. 4:11–17. 3382986 10.1093/bioinformatics/4.1.11

[7] Kushkina AI, Tovkach FI, Comeau AM, Kostetskii IE, Lisovski I, Ostapchuk AM, Voychuk SI, Gorb TI, Romaniuk LV 2013 Complete genome sequence of *Escherichia* phage Lw1, a new member of the RB43 Group of Pseudo T-Even bacteriophages. Genome Announc 1(6):e00743-13. doi:10.1128/genomeA.00743-13. PMC3861415 24336362

[8] Camacho C, Coulouris G, Avagyan V, Ma N, Papadopoulos J, Bealer K, Madden TL 2009 BLAST+: architecture and applications. BMC Bioinformatics 10:421. doi:10.1186/1471-2105-10-421. 20003500 PMC2803857

[9] Hunter S, Apweiler R, Attwood TK, Bairoch A, Bateman A, Binns D, Bork P, Das U, Daugherty L, Duquenne L, Finn RD, Gough J, Haft D, Hulo N, Kahn D, Kelly E, Laugraud A, Letunic I, Lonsdale D, Lopez R, Madera M, Maslen J, McAnulla C, McDowall J, Mistry J, Mitchell A, Mulder N, Natale D, Orengo C, Quinn AF, Selengut JD, Sigrist CJ, Thimma M, Thomas PD, Valentin F, Wilson D, Wu CH, Yeats C 2009 Interpro: the integrative protein signature database. Nucleic Acids Res. 37:D211–D215. doi:10.1093/nar/gkn785. 18940856 PMC2686546

